# Case of Small-Pox Twenty-Three Years after Vaccination; with Observations

**Published:** 1827-05

**Authors:** C. Heineken


### *Case of Small-Pox twenty-three Years after Vaccination; with Observations.* By C. Heineken, M.D.

As the following case excited considerable interest, and awakened some slumbering prejudices in the circle within which it occurred, I am induced to publish it; and I shall do so in the words of the mother of the lady to whom it refers, in order that it may escape the possibility of that taint and sophistication which even a moderate portion of professional moulding is so apt to confer; a species of tampering with a witness, to which we are indebted for most of the many "false facts" which have become a reproach and a bye-word to us. "Valeat quantum valere potest."

"My daughter was vaccinated at the age of four or five days, so long ago as the month of May, 1803. The operation was performed by Mr. Napp, a surgeon, who at that time resided in

1

Brompton-row, near Hyde Park. Dr. Poignand (who had attended me in my lying-in, and who was considered a gentleman of eminence in his profession,) saw the infant every day, and both gentlemen seemed perfectly satisfied that the vaccination had taken, and was good of its kind. Mrs. —— has several times been exposed to the contagion of the small-pox, particularly when about twelve years of age; and at school, a child of two years old, thickly covered with the small-pox, was brought into the room, and my daughter, in turn with others of the young people, took it in her arms, and nursed it on her lap.

"Twenty-three years elapsed between the time of the vaccination and that of Mrs. ——'s sickening for the small-pox: this last event happened on the 24th of last July. On the 30th, the disease first showed itself, and eleven days after (the 10th of August,) the pustules first began to mature. Six months elapsed from the introduction of the disease on the island to the period of Mrs. ——'s taking the infection; and it may be worthy of remark, that our house was surrounded by it, and immediately opposite to the church and ground where numbers were daily buried who had died of the complaint. We took your opinion in March, full three months before the time of her sickening."

In transcribing the above case, I have done so literally; and I take it for granted, upon the evidence which has been adduced, that the child had the genuine cow-pox at the age of four or five days. I know that the same child, now a woman, has just passed through, at the hazard of her life,—not a pseudo-variola, not a modified small-pox, or an exasperated varicella,—but as orthodox, old-fashioned a small-pox as any humoral pathologist could have wished for, or any friend to humanity have deprecated; and yet I acknowledge that, so far from weakening, it confirms my reliance upon the anti-variolous power of vaccination; for it proves that the introduction of that specific matter into the system at the extremely early age of four or five days, conferred immunity (and that too, in one instance at least, under circumstances of unusual exposure to the contagion,) for a period of twenty-three years! a sufficiently long term of exemption to induce the most prejudiced and sceptical to rank it amongst those exceptions which confirm the rule. It may perhaps, on the other hand, be urged by those who, in advocating the omnipotence of vaccination under all circumstances, are unwilling to allow any instance of its failure, that no evidence in detail has been adduced that the child ever had genuine cow-pock. This is certainly true: it is also true that the scar upon the arm is large, superficial, irregular, and undefined; that it wants what are generally termed its specific characters. But I am unwilling to admit that these specific characters must attach to it under all circumstances. I conceive that the permanence and depth of the cicatrix arise from its being (as all genuine cow-pox should be,) more than skin-deep; and that its round, circumscribed, marginated appearance is owing to the original boundary which, in a vigorous healthy system, was at the time of vaccination set up by a deposition of lymph into the surrounding healthy cellular membrane, to prevent its spreading: but I question very much whether we are warranted in expecting that such a process would generally be completed in an infant which had enjoyed only four, or at most five, days of independent existence. Admit, however, upon the sole, and what I consider insufficient, evidence of the scar, that two professional men of practice and experience were deceived themselves, or deceived others, and you have then the anomalous (I had almost said miraculous) circumstance of frequent exposure to, and in one instance actual and intimate contact with, a highly infectious disease during twenty-three years, with perfect impunity.

Upon a fair and impartial review of all the circumstances, I think, therefore, I am justified in saying, that the infant had the genuine cow-pox when four or five days old; that its anti-variolous power continued in force for three-and-twenty years, and then ceased; and that its character ought to be rather exalted than depressed by the fact of its having exerted its influence so long, when its introduction had taken place at a period so nearly bordering upon the incipient verge of infantile existence. The wonder is, not that it failed when it did, but that it was so long available.

I may take this opportunity of stating, that, as far as my observations and inquiries go, no other well-authenticated instance of genuine small-pox after genuine and well-authenticated vaccination has occurred here: spurious cases of both are not wanting.

Before closing this subject, I must in candour state that, when called upon for an opinion regarding the propriety of inoculating the lady in question, I stated my conviction that vaccination was, in a very large majority of cases, a complete safeguard; that I reposed the most implicit confidence on it; that although some (comparatively few) instances of its failure had occurred, yet I considered them confirming exceptions, rather than the contrary, of a general rule; that it had been stated to lose its influence after a certain, or rather uncertain, period, but that I doubted the fact. I refused, on principle, to inoculate with small-pox; and I omitted (which I now regret) to re-vaccinate: but, if ever again small-pox should prevail, I would practise myself, and advise others to adopt, the plan of re-vaccination universally.

*Funchal, Madeira; 28th December,* 1826.

